# Mediating role of depressive symptoms on the relationship between sleep duration and cognitive function

**DOI:** 10.1038/s41598-023-31357-6

**Published:** 2023-03-11

**Authors:** Liqun Wang, Shulan He, Ning Yan, Ruiping Pan, Yang Niu, Jiangping Li

**Affiliations:** 1 Department of Epidemiology and Statistics, School of Public Health and Management, Ningxia Medical University, Yinchuan, 750004 China; 2 Key Laboratory of Environmental Factors and Chronic Disease Control, Ningxia Medical University, Yinchuan, 750004 China; 3 Heart Centre &, Department of Cardiovascular Diseases, General Hospital of Ningxia Medical University, Yinchuan, 750004 Ningxia China; 4 Department of Chinese Medicine, The Second People’s Hospital of Shizuishan, Shizuishan, 753000 China; 5 Key Laboratory of the Ningxia Ethnomedicine Modernization, Ministry of Education, Ningxia Medical University, Yinchuan, 750004 China

**Keywords:** Diseases, Health care, Medical research, Risk factors

## Abstract

Although some studies have shown the association between sleep duration and cognitive impairment is positive, the mechanism explaining how sleep duration is linked to cognition remains poor understood. The current study aims to explore it among Chinese population. A cross-sectional study of 12,589 participants aged 45 or over was conducted, cognition was assessed by three measures to capture mental intactness, episodic memory, and visuospatial abilities. The Center for Epidemiologic Studies Depression Scale 10 (CES-D_10_) was administered during the face-to-face survey to assess depressive status. Sleep duration was reported by the participants themselves. Partial correlation and linear regression were used to explore the association between sleep duration, cognition, and depression. The Bootstrap methods PROCESS program was used to detect the mediation effect of depression. Sleep duration was positively correlated with cognition and negatively with depression (*p* < 0.01). The CES-D_10_ score (*r* = − 0.13, *p* < 0.01) was negatively correlated with cognitive function. Linear regression analysis showed sleep duration was positively associated with cognition (*p* = 0.001). When depressive symptoms were considered, the association between sleep duration and cognition lost significance (*p* = 0.468). Depressive symptoms have mediated the relationship between sleep duration and cognitive function. The findings revealed that the relationship between sleep duration and cognition is mainly explained by depressive symptoms and may provide new ideas for interventions for cognitive dysfunction.

## Introduction

With the rapid development of society, the prevalence of cognitive dysfunction has been gradually rising^1^ and is a crucial public health issue in China. In 2011, national figures showed that 9% of elderly individuals had cognitive impairment^2^. In 2016, a meta-analysis suggested that the pooled prevalence of mild cognitive impairment in the Chinese population had increased to 14.71%^3^. Cognitive impairment is associated with an increased risk for disability and economic burden and is an essential element of the diagnostic criteria for dementia^4,5^. Therefore, cognitive impairment is a significant burden on patients, their families, or society^6^.

Various factors affect cognitive impairment, such as ApoE4 status, smoking, hypertension, diabetes mellitus, cerebrovascular disease, and physical, intellectual, and social activities^7–9^. Furthermore, research on sleep and health has become active in recent years. Sleep duration plays a vital role in protecting individuals from cognitive decline^10^. Short sleep duration was associated with greater age-related brain atrophy and cognitive decline among old adults. Each 1-h decrease in sleep duration could predict a 0.67% greater annual decrease in global cognitive performance^11^. In addition, several studies have revealed a U-shaped association between sleep duration and cognitive impairment, and both shorter and longer sleep durations have been related to a higher risk of cognitive disorders^12–14^. However, the relationship between sleep duration and cognitive function in middle-aged and elderly individuals is still unclear.

Sleep duration has also been commonly associated with depression^15^. Several studies have reported that short sleep durations exacerbate depressive symptoms^16^. A meta-analysis indicated that short and long sleep duration was significantly associated with an increased risk of depression in adults^17^. There was a curvilinear relation between sleep duration and depression, such that both short and long sleep durations were associated with depression^18^. Moreover, severe depression was associated with a relatively faster rate of cognitive decline among middle-aged and old adults^19–21^.

The links between sleep duration, depression, and cognitive function are interrelated. Depression was shown to mediate the relationship between short sleep duration and life satisfaction^22^. What then is the relationship between sleep duration, depression, and cognitive function? The current study sought to examine the mediating effect of depression on the relationship between sleep duration and cognitive function in Chinese adults. We hypothesized that sleeping for a long enough duration is associated with better cognitive function and that this association would be mediated at least in part by depression.

## Methods

### Study sample

The data were from the China Health and Retirement Longitudinal Study (CHARLS) survey that targeted middle-aged and elderly individuals (45 + years of age) in China. The CHARLS is an ongoing follow-up study with exams performed every 2 years for a total of 3 waves from 2011 to 2015, which provided a broad range of information from demographic characteristics to health status^23,24^. The detailed sampling process can be found elsewhere^25^. In summary, the baseline survey was fielded from June 2011 to March 2012 and involved 17,705 respondents randomly selected with a probability proportional to scale (PPS) in 450 villages/resident committees, 150 counties/districts, and 28 provinces. A face-to-face interview was conducted via computer-assisted personal interviewing (CAPI) technology.

In this study, we adopted the data from wave 1, and a final sample of 12,589 respondents was included in the analysis. The inclusion criteria were as follows: (i) had complete data related to sleep duration; (ii) had complete data related to cognition measures at baseline; and (iii) had marital status, residency, smoking, drinking, body mass index (BMI), hypertension, dyslipidemia, and diabetes or high blood sugar data at baseline. The exclusion criteria were: (a) unconsciousness caused by any forms; (b) any obvious cognitive disabilities or deafness, aphasia, or other language barriers; and (c) sleep disorders and taking hypnotics, as well as some particular work needed to going to bed late.

This study was approved by the Biomedical Ethics Review Committee of Peking University (IRB00001052-11015) and the University of Newcastle Human Research Ethics Committee (H-2015-0290). All interviewees were required to sign informed consent.

### Outcome variable

Cognitive function was assessed using three measures at baseline: (a) the Telephone Interview of Cognitive Status (TICS-10); (b) word recall (WR); and (c) figure drawing (FD)^23,26^. These were used to estimate cognitive domains of mental intactness (orientation to time and attention), episodic memory, and visuospatial abilities. The overall cognitive score was calculated as the sum score of the TICS-10 (a sum of 10 scores), WR (a sum of 10 scores), and FD (a sum of 1 score) and could range from 0 to 21. The TICS-10 includes 10 items (score ranges from 0 to 10) that estimate the intactness of mental health, including orientation to time and attention^26^. The WR test assesses episodic memory and is the average number of correct immediate and delayed recalls from a list of 10 Chinese nouns^24,26^. The total score on the WR is 10. The FD test was used to measure visuospatial abilities^24^. The participants were shown two overlapping pentagons for this test and asked to draw the same picture. Scores of “1” and “0” indicate participants who successfully finished and failed the task, respectively.

### Independent variable

Sleep duration was obtained via the following question. “During the past month, how many hours of actual sleep did you get at night (average hours for one night)? (This may be shorter than the number of hours you spend in bed)”.

The Chinese version of the 10-item short form of the Center for Epidemiological Studies Depression Scale (CES-D_10_)^27^ was employed to indicate depressive symptoms, which has been validated among elderly individuals in China^28,29^. Ten items were contained in the CES-D_10_, and each item was scored from 0 to 3 in response to a 4-point Likert scale (from ‘rarely or none of the time’ to ‘most or all of the time’). The total score can range from 0 to 30, with higher scores indicating higher levels of depression. A cut-off score ≥ 10 was used to distinguish the participants who had obvious depressive symptoms^30^. A previous study showed that the CES-D_10_ scale has good reliability and validity (Cronbach’s α = 0.81)^31^.

### Other covariates

Covariates included age, sex (male vs. female), educational level (illiterate, primary school, or junior high school and above), marital status (married, cohabitating and divorced, separated, widowed, or never married), residency (rural vs. urban), BMI (continuous data), smoking, drinking, hypertension, dyslipidemia, and diabetes or high blood sugar. Smoking status was measured with the question, “Have you ever chewed tobacco, smoked a pipe, smoked self-rolled tobacco or smoked cigarettes/cigars,” and the possible answers included the following 3 options: (1) Yes; (2) No, or (3) Quit. Alcohol consumption status was measured with the question, “Did you drink any alcoholic beverages, such as beer, wine, or liquor in the past year, and if so, how often?”, and the possible answers included the following: (1) Drink more than once a month; (2) Drink but less than once a month; or (3) Do not drink. The status of hypertension, dyslipidemia, diabetes or high blood sugar was estimated with the following question: “Have you been diagnosed with the conditions listed below by a doctor?”.

### Statistical analyses

Analyses were performed using the Statistical Package for the Social Sciences (SPSS) version 24.0 (SPSS Inc., Chicago, Illinois, USA). Means and standard deviations or frequency percentages were used to describe variables. Partial correlations were performed after controlling for age, sex, education, marital status, residency, smoking, drinking, BMI, hypertension, dyslipidemia, diabetes, or high blood sugar. A linear regression model was used to examine the association between sleep duration, depressive symptoms, and cognitive function. Bootstrapping methods of PROCESS developed by *Hayes*^32^ were employed to examine the mediation effect of depressive symptoms on the relationship between sleep duration and cognitive function. In the *PROCESS* procedure, the number of models chosen was 4, the bootstrap sample was set to 5000, and the bias-corrected percentile bootstrap confidence interval was used to evaluate the effect size. If the confidence interval does not contain 0, it would indicate that the mediation effect was statistically significant^33^. Sensitivity analyses were performed using Structural Equation Modelling (SEM) approach.

### Ethics approval and consent to participate

Ethics approval for the study was granted by the Ethics Review Committee of Peking University, and all the participants provided signed informed consent at the time of participation. The study methodology was carried out in accordance with approved guidelines.

## Results

### Demographic characteristics

As shown in Table 1, approximately half of the participants were male, and approximately 1/5 were illiterate. Most (73.1%) were rural residents. Overall, 45.9% of the participants had depressive symptoms. The average age was 67.5 (SD = 9.6) years, with a range of 45 to 105 years. The average sleep duration was 6.4 (SD = 1.8) hours, the average CES-D_10_ score was 9.8 (SD = 4.7), and the average cognitive function score was 8.1 (SD = 2.8).

**Table 1:** Demographic characteristic of participants.

Variables	Total (N = 12,589)
Age, mean (SD)	67.5 (9.6)
Gender, male, n (%)	6131 (48.7)
Marital status, Married, n (%)	44 (2.6)
Education level, n (%)	
Illiterate	2832 (22.5)
Primary	5017 (39.9)
Junior	2870 (22.8)
Senior or above	1870 (14.9)
Residency, rural, n (%)	9307 (73.1)
Smoking, n (%)	4969 (39.5)
Drinking, n (%)	4171 (33.1)
BMI, mean (SD)	23.7 (4.0)
Hypertension	3099 (24.6)
dyslipidemia	1233 (9.8)
Diabetes or high blood sugar	731 (5.8)
Sleep duration	6.4 (1.8)
CES-D	9.8 (4.7)
Depressive symptoms, n (%)	2077 (16.5)
Cognitive function	8.1 (2.8)

*BMI* body mass index, *SD* standard deviation.

### Correlation analysis

**Figure 1:**
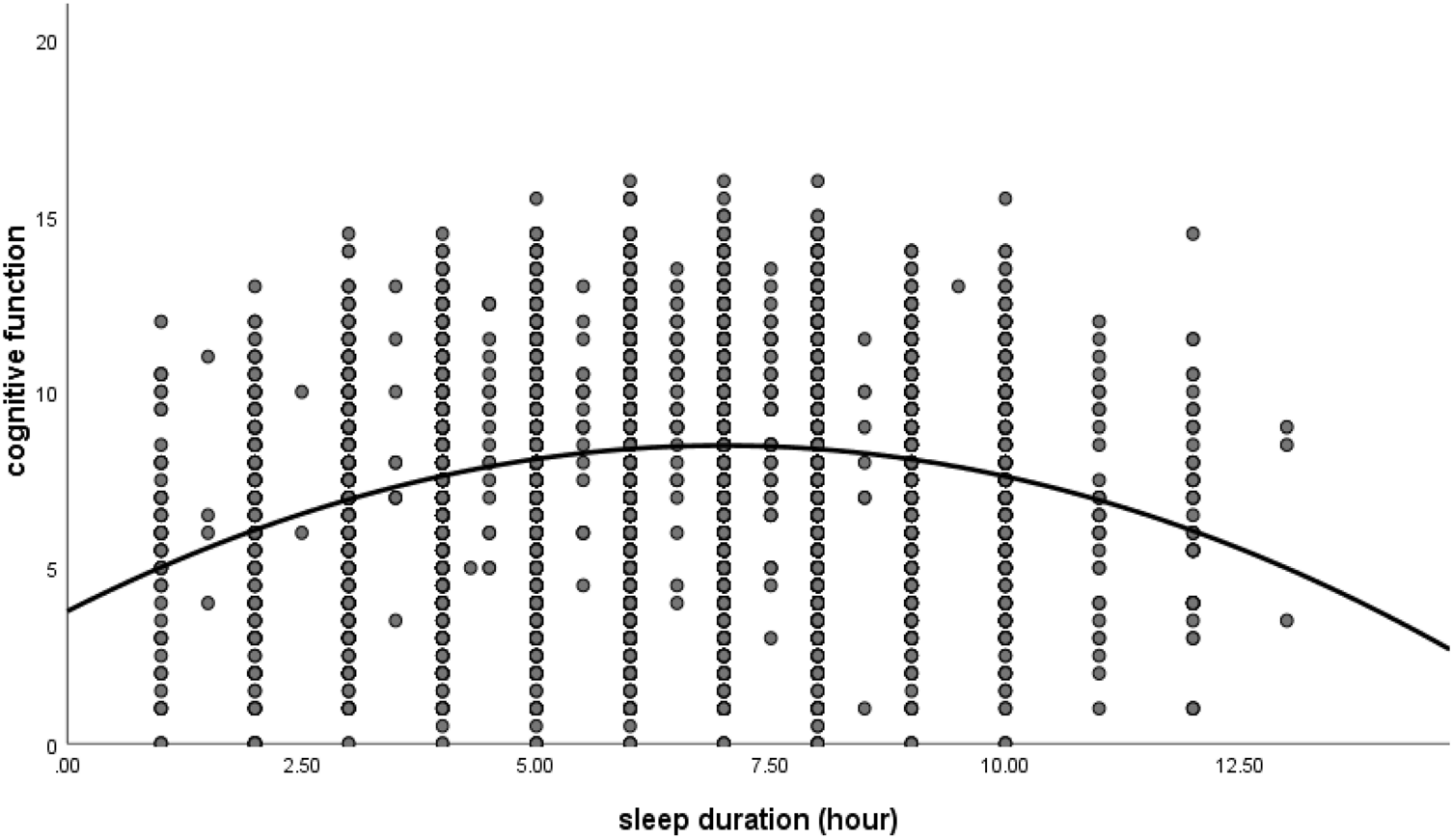
A U-shaped association between sleep duration and cognitive function.

**Figure 2:**
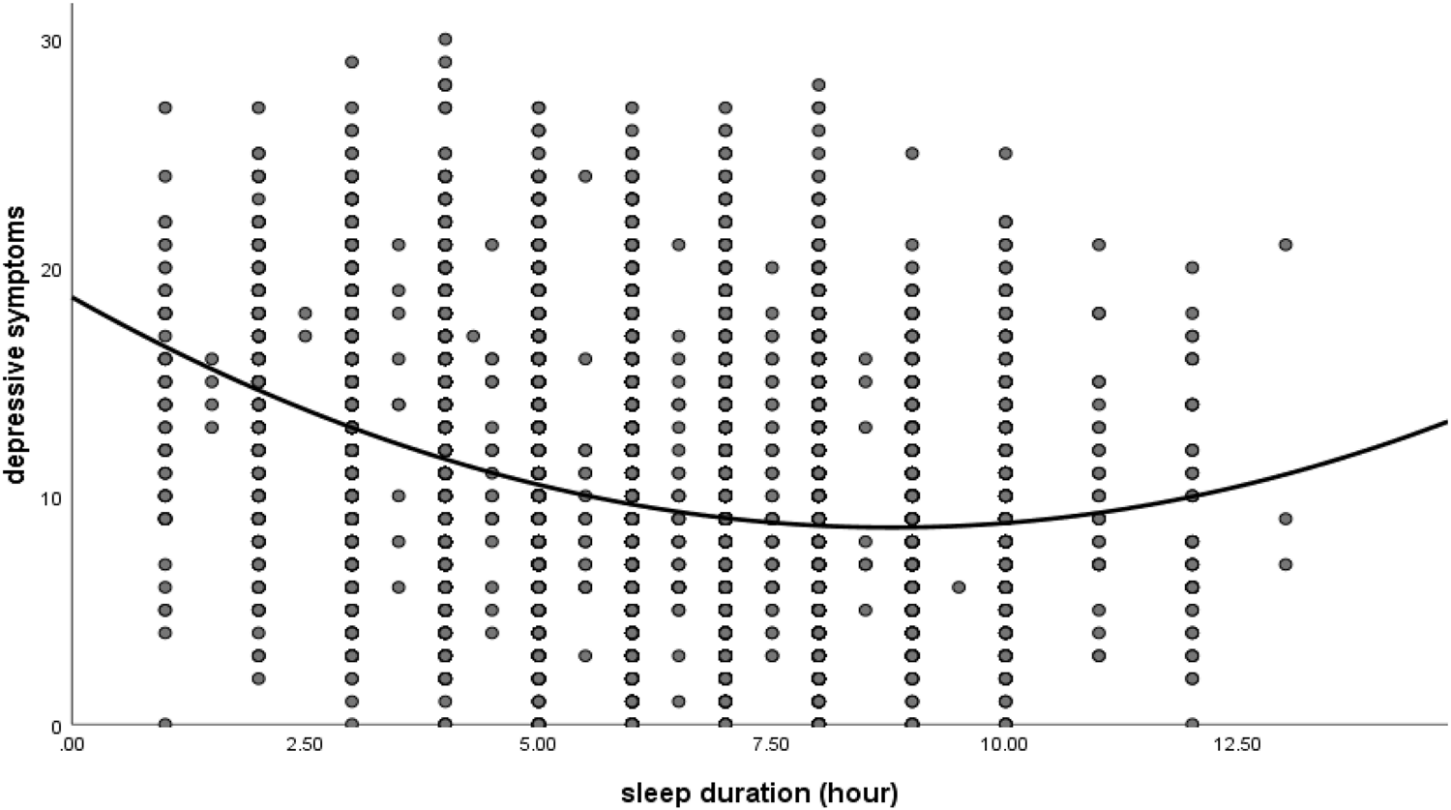
A U-shaped association between sleep duration and depressive symptoms.

After controlling for covariates, sleep duration was positively correlated with cognitive function (r = 0.03, 95% CI 0.02 to 0.05, *P* < 0.01) and inversely associated with depression scores (CES-D_10_; r = − 0.24, 95% CI − 0.26 to − 0.22, *P* < 0.01). CES-D_10_ scores were negatively correlated with cognitive function (r = − 0.13, 95% CI − 0.15 to − 0.10, *P* < 0.01), as shown in Table 2. As shown in Fig. 1, an inverted U-shaped association between sleep duration and cognitive function was found. Meanwhile, a U-shaped association between sleep duration and depressive symptoms was also found (Fig. 2).

**Table 2:** correlation matrix (n = 12,589).

Variables	Mean	SD	Sleep duration	CES-D	Cognitive function
Sleep duration	6.4	1.8	1		
CES-D	9.8	4.7	− 0.24**	1	
Cognitive function	8.1	2.8	0.03**	− 0.13**	1
Effect size	0.03				

*SD* standard deviation, *CES-D* the Center for Epidemiologic Studies Depression Scale.

All the analysis controlled for age, gender, education, marital status, residency, smoking, drinking, BMI, hypertension, dyslipidemia, and diabetes or high blood sugar.

**p < 0.01, *p < 0.05.

### Linear regression analyses

As shown in Table 3, sleep duration was positively correlated with cognitive function (Model 1). When depressive symptoms were added in Model 2, the association between sleep duration and cognitive function lost significance (β = − 0.06, 95% CI − 0.22 to 0.10, *P* = 0.468).

**Table 3:** Linear regression between depression, sleep duration and cognitive function.

Variables	Model 1		Model 2	
	*P value*	*β (95% CI)*	*P value*	*β (95% CI)*
Age	< 0.001	− 0.05 (− 0.06, − 0.05)	< 0.001	− 0.05 (− 0.06, − 0.05)
Gender	0.026	0.15 (0.02, 0.29)	0.199	0.09 (− 0.05, 0.22)
Education	< 0.001	1.10 (1.05, 1.16)	< 0.001	1.09 (1.03, 1.14)
Marital status (married)	0.012	0.19 (0.04, 0.34)	0.046	0.15 (0.01, 0.30)
Rural	< 0.001	− 0.80 (− 0.92, − 0.68)	< 0.001	− 0.76 (− 0.88, 0.24)
Smoking	0.079	0.11 (− 0.01, 0.24)	0.066	0.12 (− 0.01, 0.24)
Drinking	0.068	− 0.10 (− 0.21, 0.01)	0.039	− 0.12 (− 0.23, − 0.01)
BMI	< 0.001	0.01 (0.04, 0.06)	< 0.001	0.04 (0.03, 0.06)
Hypertension	0.608	− 0.03 (− 0.15, 0.08)	0.992	− 0.01 (− 0.12, 0.11)
dyslipidemia	0.127	0.13 (− 0.10, 0.30)	0.077	0.15 (− 0.02, 0.31)
Diabetes or high blood sugar	0.592	0.05 (− 0.15, 0.26)	0.394	0.09 (− 0.11.0.29)
Depression	NA	NA	< 0.001	− 0.54 (− 0.65, − 0.43)
Sleep duration	0.001	0.04 (0.02, 0.07)	0.468	− 0.06 (− 0.22, 0.10)
Sleep duration × depression	NA	NA	0.350	− 0.10 (− 0.31, 0.11)
*R*^*2*^	0.288		0.298	

*BMI* body mass index, *95% CI* 95% confident interval.

### Mediation

For this analysis, sleep duration was an independent variable, depressive symptoms were the mediator variable, and cognitive function was a dependent variable. After controlling for age, sex, education, marital status, residency, smoking, drinking, BMI, hypertension, dyslipidemia, and diabetes or high blood sugar, a mediation model was conducted. The total effect, direct effect, and mediating effect of sleep duration on cognitive function with standard errors and confidence intervals were obtained using the bootstrap method. The results showed that the direct effect was non-significant, while the total effect and the indirect effect were significant, indicating that depressive symptoms fully mediated the relationship between sleep duration and cognitive function. The mediation effect of depressive symptoms on the relationship between sleep duration and cognitive function accounted for 93.0% (0.040/0.043) of the total effect (Table 4). The bias-corrected program test results indicated that the 95% confidence interval was 0.033 to 0.047, a range that did not include 0, indicating that the mediation path was statistically significant. Considering the possible relationship between age and depressive symptoms, the exploring analysis conducted stratified (use a cutoff point of 60 years old) by age showed in Table 5. The mediation effect of depressive symptoms on the relationship between sleep duration and cognitive decline persists in those who < 60 and ≥ 60 years old.

**Table 4:** The Mediating effect of depression on the relationship between sleep duration and cognitive function.

Effect	*β*	*SE*	*P* value	Bias-corrected *95% CI*	
				Lower	Upper
Total (n = 1742)					
Total effect	0.043	0.013	0.001	0.017	0.068
Indirect effects (mediating effect)	0.040	0.004	< 0.001	0.033	0.047
Direct effects	0.003	0.013	0.836	-0.023	0.029

*SE* standard error, *95% CI* 95% confident interval.

All the analysis under controlling of age, gender, education, marital status, residency, smoking, drinking, BMI, hypertension, dyslipidemia, and diabetes or high blood sugar.

**Table 5:** The mediation model of depressive symptoms stratified by age^a^.

Effect	Bias-corrected 95% CI				
	*β*	*SE*	*P*-value	Lower	Upper
Age < 60 years old					
Total effect	0.087	0.286	0.002	0.031	0.144
Indirect effects	0.042	0.008	< 0.001	0.028	0.059
Direct effects	0.045	0.029	0.119	-0.011	0.102
Age ≥ 60 years old					
Total effect	0.043	0.015	0.004	0.014	0.072
Indirect effects	0.037	0.021	< 0.001	0.029	0.046
Direct effects	0.006	0.015	0.700	-0.240	0.036

^a^The effect adjusted the covariates include gender, education, marital status, residency, smoking, drinking, BMI, hypertension, dyslipidemia, and diabetes or high blood sugar.

### Sensitivity analysis

**Figure 3:**
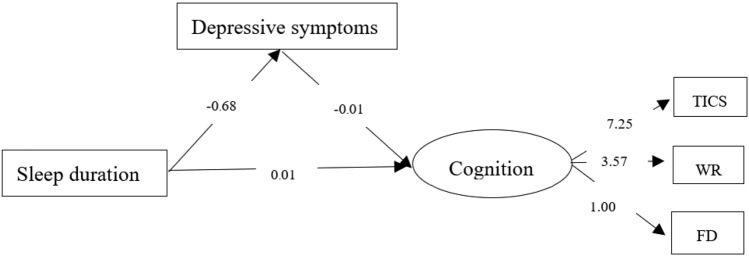
The mediation model of depressive symptoms in the relationship between sleep duration and cognitive function. *TICS* the telephone interview of cognitive status, *WR* word recall, *FD* figure drawing.

As showed in Fig. 3, the SEM approach was conducted to sensitivity analysis. The results showed sleep duration was positively associated with cognitive function and inversely related to depressive symptoms, and depressive symptoms were negatively associated with cognitive function. These results were consistent with the binary correlation results even though the pathway coefficient was not the same due to the different methods. This validated the methodological robustness of the findings.

## Discussion

The current study explored the association between sleep duration and cognitive function and the mediating effect of depressive symptoms on this relationship among middle-aged and elderly individuals in mainland China. In this study, we found that a longer enough sleep duration (in our population, only 956 (7.5%) participants reported sleeping ≥ 9 h per night) was associated with a reduced risk of cognitive decline, and these beneficial effects were mediated by depressive symptoms. The mediation effect accounted for 93.0% of the total effect.

Sleep duration was positively associated with cognitive function scores, and these data implied that short sleep duration might be a risk factor for cognitive impairment, consistent with a study showing that women sleeping for short durations had worse global cognition than those sleeping longer^34^. Short sleep duration was also reported to be a risk factor for cognitive impairment^11,35^. We speculate that the mechanism may be that short sleep duration has been related to the β-amyloid (Aβ) burden, which is one of the hallmarks of Alzheimer’s disease^36^. In addition, shorter sleep durations were associated with a greater degree of age-related brain atrophy^11^. However, our results were conflicted with prior research indicating that long sleep durations have been associated with cognitive impairment^37,38^. They explained this phenomenon by the fact that increased sleep fragmentation is associated with decreased cognitive performance and that longer sleep durations may result in more frequent nighttime awakenings or more sleep-in-bed time^39^. It should be noted that the r-value between sleep duration and cognitive function is somehow very small (r = 0.03); this maybe because there existed an inverted-U-shaped relationship between sleep duration and cognitive function. Previous research has reported that short or long sleep durations were significantly associated with memory impairment, might be a key marker for increased risk of cognitive impairment, and showed a U-shaped association^14^, which further confirmed that sleeping for enough duration was essential for maintaining cognitive function.

Furthermore, our results showed that depressive symptoms mediated the relationship between sleep duration and cognitive function. Previous studies have confirmed that depressive symptoms mediated the relationships between sleep duration and several factors, such as life satisfaction^22^. The present findings are also supported by a study showing that depression mediated the relationship between religiosity and cognitive impairment in older adults^40^. The relationships between sleep duration and depression have undoubtedly attracted people’s attention. The current study found that sleep duration was inversely associated with depression and implied that short sleep duration may be a risk factor for depression. This was consistent with a previous study that revealed that poor sleep, including short sleep duration, was independently associated with depression^41^. A meta-analysis also indicated that short sleep duration was linked to an increased risk of depression among adults^16^. Furthermore, we also found a U-shaped relationship between sleep duration and depressive symptoms, which is consistent with a previous study, that reported that insufficient or prolonged sleep was independently associated with depressive symptoms in middle-aged and elderly people, showing a U-shaped relationship^42^. Our study found that CES-D_10_ scores were negatively associated with cognitive function scores; that is, depression was positively associated with an increased risk of cognitive impairment. Previous studies have also suggested that depression was positively associated with cognitive impairment^43,44^. Furthermore, depression in elderly patients appears to predict dementia strongly^45^. These results might indicate that short sleep durations are associated with cognitive function through the mediating role of depression. In light of previous research, possible reasons are that shorter sleep duration may lead to daytime tiredness, which may lead to increased negative events and emotions, which may lead to depression^46^.

By the way, it was found that 45.9% of the participants had depressive symptoms. This significantly exceeds the data on the prevalence of depression in the general population. It may be that the percentage of depressive symptoms reported by previous studies were different because of the different participants, methods, and design or different measurement scales, etc. for example, the results from a systematic review and meta-analysis showed that the summary prevalence of depression or depressive symptoms among medical students estimates ranged across assessment modalities from 9.3% to 55.9%^47^. The prevalence of depressive symptoms among Chinese rural residents was 34.0%^48^. In addition, in our study, we defined the variable as depressive symptoms not depression when using the CES-D scale (CES-D score ≥ 10). Another study also reported that a large sample of Tibetan people with depressive symptoms (scores ≥ 8) accounted for 52.3% of the total sample, and participants with depression (scores ≥ 14) accounted for 28.6%^49^. In addition, the average sleep duration for the Chinese population was 6.4 (SD = 1.8) hours. It looks less than the recommended and differs from the data on the habitual sleep time from Europe or the United States. In our study, the average age of participants was 67.5 (SD = 9.6) years. The old adults usually had short sleep duration. A study showed that participants aged 63.3 slept ≤ 7 h accounted for 84.6%^50^. Besides, it may be the characteristics of the population sample.

Given the increasing proportion of aged individuals and increasing prevalence of dementia in the Chinese population, the strength of the present findings is its relevance for using large-sample data sets to understand the mechanisms underlying the effects of sleep duration on cognitive function, which may stimulate further research. Several limitations existed in the present study. First, this was a cross-sectional analysis, and causal inferences were not possible. Hence, a future study with a longitudinal design would be necessary to determine causal relationships. Second, sleep duration was collected based on self-reporting, which may involve information bias despite common use in epidemiological studies due to feasibility considerations. Meanwhile, the current study does not determine the cause/nature of short sleep duration, which may include sleep apnea, pain, or other sleep-related disorders. Third, a limited number of neuropsychological test variables were used in this study, as the three tests do not encompass most cognitive domains, which may affect the results.

## Conclusion

In conclusion, this study provides evidence for the relationship between sleep duration and cognitive function among middle-aged and elderly individuals, suggesting a possible mechanism to explain how sleep duration is related to reduced cognition in studies examining that association. We found that depressive symptoms may mediate the relationship between sleep duration and cognitive impairment. Hence, in the clinic, clinicians may suggest that patients ensure that they get enough sleep and maintain a good mood. Clinicians may use melatonin to improve the sleep quality of those experiencing cognitive dysfunction and can also consider cognitive-behavioral therapy.

## Supplementary Information

Supplementary Information.

## Data Availability

The datasets generated and/or analyzed during the current study are available in a Supplementary File.

## Abbreviations

CES-D: The center for epidemiologic studies depression scale
TICS: The telephone interview of cognitive status
WR: Word-recall
FD: Figure-drawing
